# Optimizing irrigation strategies in wolfberry-alfalfa intercropping system in arid saline-alkali region: impacts on crop physiology, yield formation and quality parameters

**DOI:** 10.3389/fpls.2025.1607461

**Published:** 2025-05-20

**Authors:** Haiyan Li, Yanbiao Wang, Yuanbo Jiang, Bojie Xie, Guangping Qi, Minhua Yin, Yanxia Kang, Yanlin Ma, Yayu Wang, Boda Li, Wenjing Chang

**Affiliations:** ^1^ College of Water Conservancy and Hydropower Engineering, Gansu Agricultural University, Lanzhou, China; ^2^ College of Horticulture, Gansu Agricultural University, Lanzhou, China

**Keywords:** water regulation, multicriteria assessment, water conservation, intercropping system, crop growth characteristics

## Abstract

Soil salinization caused by water scarcity in Northwest China severely limits agricultural sustainability. A forest-grass intercropping system combined with water regulation strategies can optimize soil and water resource use, reduce agricultural water stress, mitigate soil salinization, and promote sustainable and eco-efficient agricultural development in arid regions. In this study, based on a 3-year field experiment, four water regulation strategies were set up [upper and lower soil moisture limits were controlled by soil moisture content as a percentage of field water holding capacity *θ_f_*, and full irrigation W0 (75%–85%*θ_f_*), mild water deficit W1 (65%–75%*θ_f_*), moderate water deficit W2 (55%–65%*θ_f_*), and severe water deficit W3 (45%–55%*θ_f_*)], the effects of water regulation on crop growth, physiology, yield and quality in wolfberry-alfalfa system were analyzed. The results showed that (1) increasing water deficit would limit the growth and development of wolfberry and alfalfa, with wolfberry and alfalfa growth being maximal at the vegetative growth stage-full flowering stage, and alfalfa plant height and stem thickness both being maximal in the first crop. (2) With the increase of water deficit, the chlorophyll content and photosynthetic characteristics of crops showed a decreasing trend. Among them, the daily dynamics of leaf photosynthesis showed that the net photosynthetic rate (P_n_) and transpiration rate (T_r_) of wolfberry presented a single-peak curve, the P_n_, T_r_ and stomatal conductance (Cond) of alfalfa presented a double-peak curve, and the inter-cellular carbon dioxide concentration (C_i_) of both of them reached the minimum from 12:00 to 14:00. (3) With the increase of planting years, the dry fruit yield of wolfberry and the total yield of alfalfa showed an increasing trend, while the crop quality all showed a decreasing trend; higher irrigation (W0 and W1) was more favorable to the crop yield and quality improvement. The multicriteria assessment showed that the W1 (65%–75%*θ_f_)* treatment improved crop yield and quality in the wolfberry-alfalfa intercropping system while ensuring efficient water use. This treatment serves as a water control model for water conservation, yield increase, and quality improvement in the arid saline zone and similar ecological zones of northwestern China.

## 1 Introduction

In recent years, economic development and population growth have further exacerbated the demand for freshwater resources, and the frequent occurrence of localized and regional droughts has led to the increasingly serious problem of soil salinization, posing a serious threat to global food security and ecological safety (Bai et al., 2022). According to statistics, the total area of salinized soils in the world is 1.381 billion ha, accounting for 10.7% of the total land area (FAO et al., 2024). Soil salinization, as a major form of soil degradation in arid regions, constrains crop production and seriously affects ecosystem functioning (Perri et al., 2022). Therefore, it is necessary to rationally optimize water regulation strategies in water-scarce arid and semi-arid regions in order to achieve synergistic development of crop productivity enhancement and efficient use of water resources. Wolfberry (*Lycium barbarum* L.) is a perennial plant of the Solanaceae family, which has pharmacological effects such as enhancing immunity and anti-aging, as well as strong ecological adaptability such as drought resistance and salinity tolerance, and is an excellent tree species for windbreak and sand fixation in arid and semi-arid areas (Peng et al., 2021; Wang et al., 2024). As a perennial legume forage grass, alfalfa (*Medicago sativa* L.) is high-yielding, high-quality and well-adapted, and it can absorb deep water and fix nitrogen to improve the soil and reduce the salinity epilimnion through the deep root system (Lu et al., 2024b; Tlahig et al., 2024). The intercropping of alfalfa with wolfberry can not only fully utilize the soil water and nutrient resources, but also improve the overall salt tolerance and productivity of the system through the complementary effect between plants (Lu et al., 2024a). While adapting to the special environment of arid saline areas, the wolfberry-alfalfa intercropping system provides a synergistic solution for sustainable agricultural development and ecological restoration, which is of great economic and ecological significance (Wang et al., 2024).

Water is one of the most important factors affecting plant growth, root morphology structure and distribution characteristics, and an indispensable medium for plant metabolism (Wang et al., 2024). Scientific water management can optimize the distribution pattern of photosynthetic products among different organs by regulating the dynamic balance between nutrient growth and reproductive growth of crops (Zhong et al., 2019; Zhou et al., 2022), especially by facilitating the directional translocation of assimilated products to the seeds (Xia et al., 2022). Thus, the economic yield of the crop was significantly increased. It was shown that irrigation at 80% Fc increased chlorophyll content, net photosynthetic rate and transpiration rate of crop leaves in an apple-soybean intercropping system (Wang et al., 2023a). In olive-pasture intercropping system, it was found that mild water stress stimulated the increase of total root length and biomass of monocropped olive root system, while severe drought stress significantly reduced root morphology parameters such as total root length, mean diameter, total surface area, and total volume, but intercropping with pasture alleviated the inhibitory effect of drought stress on olive root growth (Chen et al., 2020). Appropriate water regulation can also improve the quality of intercropping system crops without yield reduction or with less yield reduction. It was shown that under mild water deficit conditions in the early stage (1500 m^3^·ha^−1^ irrigation at each of the 6-leaf, pollen-dispersal, and grouting stages of maize), the population forage yields of corn-legume intercropping system were not significantly different from those of fully irrigated, but the population crude protein and crude fat contents, water-use efficiency, and relative productivity of irrigation were higher than those of fully irrigated (Wang et al., 2023b). In the faba bean-wheat intercropping system, wheat K^+^ and Na^+^ and glycine-betaine contents were higher under mild water deficit conditions (80% Fc) than under severe water deficit (40% Fc) (Cheto et al., 2024). It was also noted that apple new shoot length, soybean plant height and leaf area index, and both net photosynthetic rate and leaf photosynthetic efficiency were greatest at the fully irrigated level (80% Fc) in an apple-soybean intercropping system (Zheng et al., 2021). In addition, in the apple-maize system, the net photosynthetic rate of maize gradually increased with increasing irrigation, while the transpiration rate tended to decrease (Zhou et al., 2019). It can be seen that there are some differences in the conclusions of the studies on the effects of water regulation on the crops in intercropping systems, and further research is still needed.

Northwest China is deeply inland, with little rainfall, average annual precipitation is generally less than 400 mm (locally less than 100 mm), and evapotranspiration far exceeds precipitation, resulting in an extreme scarcity of water resources (Raza et al., 2021). Long-term drought stress makes agriculture highly dependent on irrigation, and irrational irrigation methods (e.g., flood irrigation) will further exacerbate soil salinization (Zhang et al., 2022), resulting in declining soil fertility, degradation of arable land, and severely limiting the sustainable development of local agriculture. As a planting pattern with both economic and ecological benefits in Northwest China (Wang et al., 2024), the wolfberry-alfalfa intercropping system improves the efficiency of land resource utilization and provides an effective solution for the sustainable development of agriculture in arid and saline areas. Therefore, this study took the wolfberry-alfalfa intercropping system as the research object, aiming to (1) quantify the effects of water regulation on crop growth, physiology, yield and quality in the wolfberry-alfalfa intercropping system; (2) clarify the relationship between the growth and development, physiological metabolism, and production of wolfberry and alfalfa in the intercropping system; (3) obtain the suitable water regulation model of wolfberry-alfalfa intercropping system, which will provide a reference basis for the efficient utilization of water resources in arid and saline areas.

## 2 Materials and methods

### 2.1 Description of the experimental site

**Figure 1:**
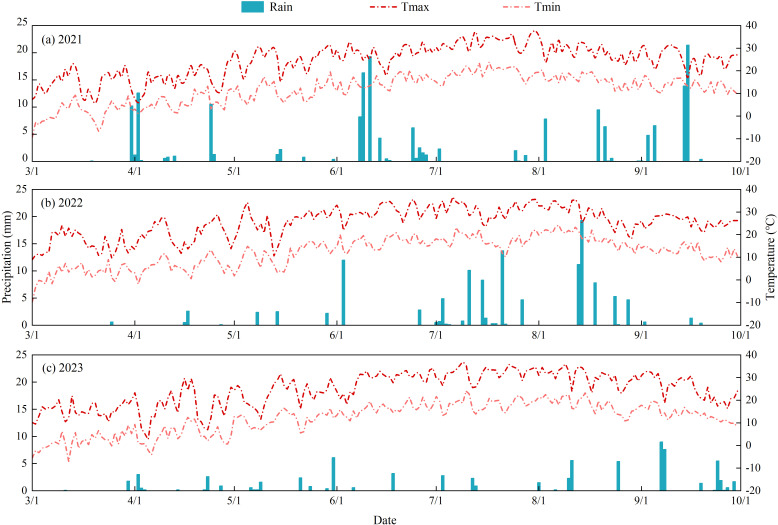
Daily distribution of precipitation and temperature during the experiment. **(a–c)** represent the daily precipitation and temperature from 2021 to 2023. T_max_ is the highest temperature of the day and T_min_ is the lowest temperature of the day.

The experiment was carried out from April to October 2021–2023 at the Irrigation Experiment Station of the Jingtaichuan Electric Power Irrigation Water Resource Utilization Center, Gansu Province (37°23′N, 104°08′E). The area has a temperate continental climate with an average elevation of 1572 m. The average multi-year temperature was 8.6°C, sunshine hours 2652 h, radiation 6.18×10^5^ J·cm^−2^, evapotranspiration 2761 mm, precipitation and frost-free period 201.6 mm and 191 d, respectively. The soil type of the test site was sandy loam, with a dry soil capacity of 1.63 g·cm^−3^, a field water holding capacity of 24.10% (mass water content), and a pH value of 8.11. The 0–60 cm soil layer of the experimental site contained total nitrogen 1.62 g·kg^−1^, total phosphorus 1.32 g·kg^−1^, total potassium 34.03 g·kg^−1^, quick-acting nitrogen 74.51 mg·kg^−1^, quick-acting phosphorus 26.31 mg·kg^−1^, and quick-acting potassium 173 mg·kg^−1^. Meteorological data were determined by the Davis small smart agrometeorological station, and the 3-year rainfall and average temperatures during the experiment were 83.93 mm and 20.49°C (2021), 118.00 mm and 20.62°C (2022), and 91.05 mm and 20.43°C (2023, **Figure 1**), respectively.

### 2.2 Experimental design and field management

**Figure 2:**
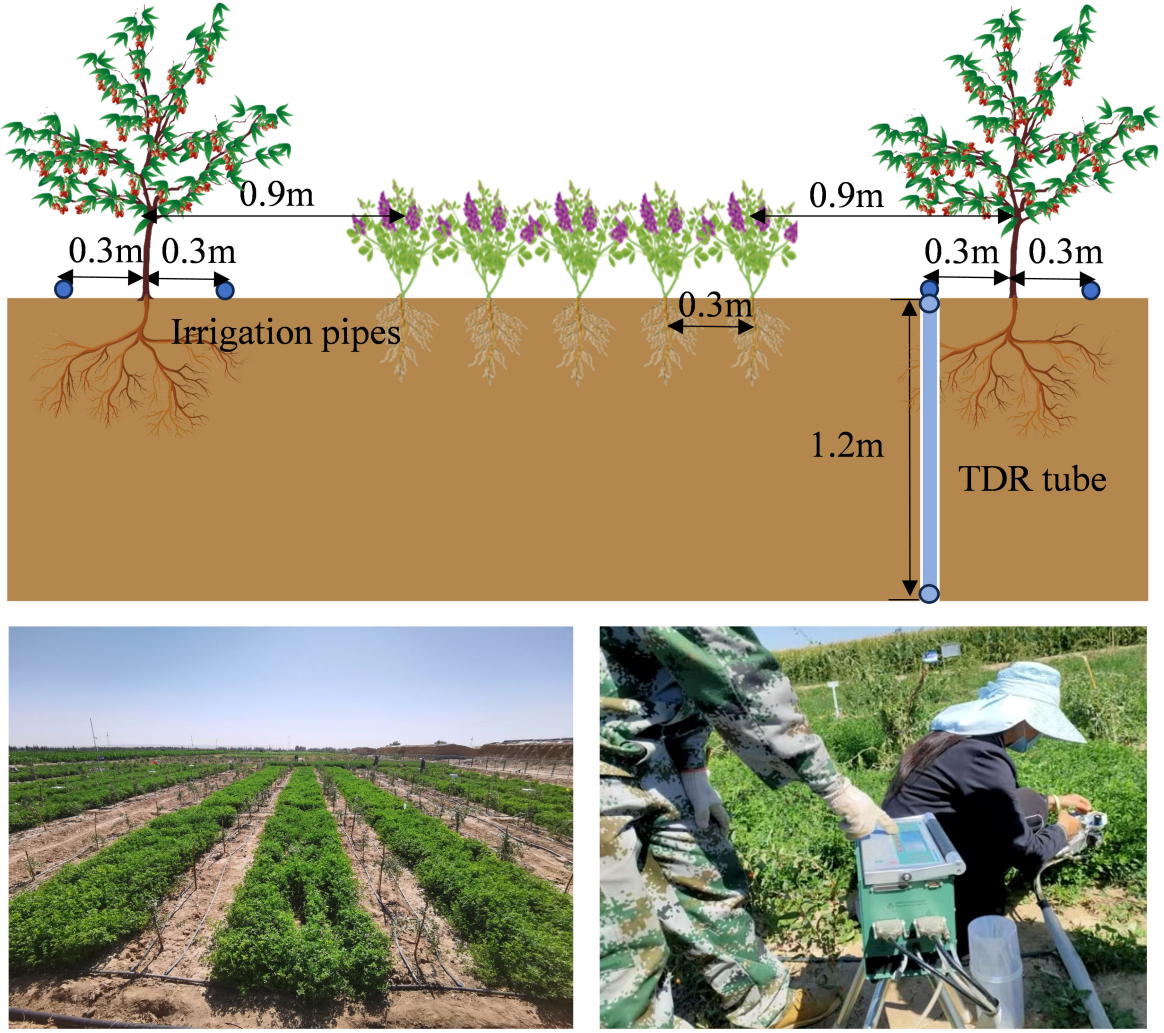
Schematic diagrams of the experimental layout.

The intercropping system of wolfberry and alfalfa was taken as the research object. The wolfberry seedling (biennial) variety was “Ningqi No.5”, plant row spacing is 1.5×3 m, and it was transplanted on April 6, 2021, with a transplanting depth of 30 cm. The alfalfa variety was “Longdong alfalfa”, which was artificially sown in drill with a sowing amount of 13 kg·ha^−1^ and a drill sowing interval of 0.3 m. The seeding depth was 30 mm, and the sowing time was April 6–7, 2021. The area of the plot was 10.2×7.5 m^2^. 4 rows of wolfberry were planted in each plot, with 5 trees in each row. Alfalfa planting rows were planted at a distance of 0.9 m from wolfberry trees (**Figure 2**). The test plots were mechanically deep ploughed one week before sowing (to ensure level ground and even irrigation) and weeds and debris were removed manually. According to the 3-year crop growth rule, the growth stage of wolfberry was divided into 5 stages (Wang et al., 2024): germination stage (4.20–5.25), vegetative growth stage (5.26–6.9), full flowering stage (6.10–7.14), peak fruit stage (7.15–8.17) and autumn fruit stage (8.18–9.15). Alfalfa was cut at the first flowering stage (When 10% of the alfalfa is in flower, it is an indication that the alfalfa is entering the first flowering stage (Liu et al., 2020)) of the first crop (6.15–6.20), the second crop (7.20–7.25) and the third crop (9.15–9.20).

Referring to the previous study (Tian et al., 2023) and local farmers’ management experience, the experiment was set up with four water regulation modes (the depth of the wet layer of the irrigation scheme was 60 cm, the upper and lower limits of soil water were measured in terms of the percentage of the field water holding capacity *θ_f_*, and irrigation was started when the soil water content dropped to the lower limit of the percentage of *θ_f_* corresponding to each treatment, and the upper limit of irrigation was the upper limit of the design interval of each treatment): full irrigation W0, 75%–85%; mild water deficit W1, 65%–75%; moderate water deficit W2, 55%–65%; and severe water deficit W3, 45%–55%, with four treatments and three replications for each treatment. A 1 m isolation zone was set between each treatment (to minimize the effect of water transport). The drip irrigation water and fertilizer integration method was used, with drip irrigation belts (Beijing Luckrain Plastic Industry Ltd., Beijing, China) laid on both sides of each row of wolfberry trees in the plot. The system configuration maintained 0.6 m arrangement spacing between belts, using drip heads with a flow rate of 2.0 L·h^−1^. Valves and water meters (precision 0.0001 m^3^) were installed in each plot to control the amount of irrigation water. Nitrogen fertilizer was used as urea (containing 46% nitrogen) at 300 kg·ha^−1^ (pure amount), which was applied during the nutritive growth period, flowering and fruiting period at the total N fertilizer distribution ratio (6:2:2); phosphorus fertilizer (Ca(H_2_PO_4_)_2_, containing 12% phosphorus) and potash fertilizer (K_2_SO_4_, containing 60% potash) in the annual Chinese wolfberry sprouting period of a one-time application, are 130 kg·ha^−1^ (pure amount). Regular weeding was carried out in the wolfberry field to eliminate the influence of weeds on the experiment, in addition, field management measures such as wolfberry pruning and crop pest control were kept in line with local cultivation practices.

### 2.3 Indicators and methods for measurement

#### 2.3.1 Soil water content

Soil water content was monitored at depths of 0–20, 20–40, 40–60, 60–80 and 80–100 cm from the trunk of wolfberry trees at 7-day intervals using a PICO-BT TRIME-TDR instrument (IMKO, Germany) (**Figure 2**). Additional measurements were taken before and after irrigation and rainfall, and corrections were made periodically using the drying method.

#### 2.3.2 Water consumption

Water consumption (Nie et al., 2019) during the growth period of the wolfberry was calculated using the water balance method.


(1) $$\text{ET}=\text{P}+{\text{W}}_{2}-{\text{W}}_{1}+I+\text{H}-\text{R}-{\text{D}}_{P}$$

where *ET* is the water consumption during the reproductive period (mm); *P* is the rainfall during the reproductive period of the crop (mm); *W*_2_ is the soil water storage in the 0–120 cm soil layer at the end of the experiment (mm); *W*_1_ is the soil water storage in the 0–120 cm soil layer at the beginning of each year’s experiment (mm); *I* is the amount of irrigation water (mm); *K* is the amount of groundwater recharge (mm); *R* is the runoff flow rate (mm); and *D_p_* is the amount of deep seepage (mm). Since the depth of groundwater in the test area is below 5 m, the terrain is flat and the amount of single rainfall is small, so *K*, *R* and *D_p_* are negligible.

#### 2.3.3 Growth indicators

(1) Wolfberry plant height, crown width and stem diameter growth. Each plot randomly selected the growth of basically the same 3 plants of wolfberry for planting and tagging, respectively, at the end of each fertility period of wolfberry using tape measure to determine the plant height (cm), wolfberry crown width (cm), vernier calipers to determine the stem diameter (mm), the difference between the two that is, the growth of wolfberry plant height (cm), crown width growth (cm) and stem diameter growth (mm).

(2) Alfalfa plant height and stem thickness. Plant height and stem thickness were measured in each alfalfa crop before mowing (early flowering stage). Nine alfalfa plants with similar growth at different locations were randomly selected from each plot, and the plant height (cm) was measured with a steel tape measure, and the stem thickness (mm) was measured with a vernier caliper at 5 cm from the ground.

#### 2.3.4 Physiological indicators

(1) Wolfberry leaf chlorophyll content (SPAD) and photosynthetic indexes. At the end of each fertility period of wolfberry, 3 complete and healthy wolfberry leaves were randomly selected from the listed and labeled wolfberry trees in each plot, and a portable chlorophyll meter (Model SPAD-502, Konica Minolta, Japan) was used to determine the SPAD of the leaves. Photosynthetic characteristics (net photosynthetic rate (P_n_, μmol·m^−2^·s^−1^), transpiration rate (T_r_, mmol·m^−2^·s^−1^), stomatal conductance (Cond, μmol·m^−2^·s^−1^), intercellular carbon dioxide concentration (C_i_, μmol·mol^−1^)) of wolfberry leaves were measured by photosynthesis meter (LI-6400 type, LI-COR, USA) for 3 consecutive days in wolfberry fruiting period to determine the daily changes of photosynthetic characteristics of wolfberry leaves. The time of measurement was from 8:00-18:00, and the measurement was made once every 2 hours.

(2) Alfalfa leaf SPAD and photosynthetic indexes. Before each crop of alfalfa was mowed (early flowering stage), 9 alfalfa plants with similar growth in different locations were randomly selected in each plot, and 3 healthy leaves were selected from each plant to determine the SPAD of leaves. The determination of alfalfa’s photosynthetic characteristics was carried out at the same time with wolfberry, and the leaf selection was the same as the determination of chlorophyll content, and the other determination of photosynthetic indexes was the same as that of wolfberry.

#### 2.3.5 Yield

(1) Wolfberry yield. As the wolfberry has not yet hung fruit in 2021, its production was not counted. The wolfberry dry fruit production in 2022 and 2023 was calculated according to the cumulative production of multiple crop harvesting. Since the wolfberry entered the fruiting period, each plot selected 3 wolfberry trees with consistent growth for harvesting, picking every 7–9 days, picking, dewaxing and drying with a single wolfberry tree as a unit, weighing method to determine the dry fruit yield of a single plant, and converted into unit area yield (kg·ha^−1^).

(2) Alfalfa yield. The yield of alfalfa was determined at the early flowering stage, 2 crops of alfalfa were mowed in 2021, and 3 crops were mowed in 2022 and 2023, with a stubble height of 5 cm. 3 sample squares of 1 m×1 m were randomly selected in each plot, and their fresh weight was weighed after mowing. Afterwards, the samples were transferred to an oven at 105°C for 30 min, dried at 75°C until constant weight, and weighed dry after cooling to calculate the hay yield (kg·ha^−1^).

#### 2.3.6 Quality indicators

(1) Wolfberry quality. 50 g of evenly mixed dried wolfberry fruits were randomly selected, total sugars content (g·100g^−1^) was determined by spectrophotometer method, polysaccharides content (g·100g^−1^) by anthrone-sulfuric acid method, amino acids content (%) by ninhydrin solution color development method, ash content (%) by direct ashing method, proteins content (g·100g^−1^) by Caulmers Brilliant Blue G-250 staining method, fats content (g·100g^−1^) by SOX606 Soxhlet extraction method, vitamin C content (mg·100g^−1^) by xylene extraction colorimetric method, and flavonoids (mg·100g^−1^) and total phenols content (mg·g^−1^) by spectrophotometric method (Zhang et al., 2024).

(2) Alfalfa quality. 100 g of dried and crushed alfalfa samples passed through 0.42 mm sieve were taken, and the acidic and neutral detergent fiber contents (%) of the forage were determined by Van Soest detergent fiber analysis using a semi-automatic fiber analyzer (F800, China). Plant nitrogen content and crude proteins content (%) were determined using a fully automatic Kjeldahl nitrogen analyzer (K1160, China), crude ash content (%) by direct ashing, and ether extract content (%) by SOX606 Soxhlet extraction (Jiang et al., 2022b). The relative feeding value is calculated as:


(2) $$\text{V}=(120/{\text{V}}_{1})\times (88.9-0.799{\text{V}}_{2})/1.29$$

Where *V* is the relative feeding value; *V*_1_ is the alfalfa neutral detergent fiber content (%); *V*_2_ is the alfalfa acid detergent fiber content (%).

#### 2.3.7 Entropy weight–multicriteria assessment

(1) Entropy weight method of weighting:

Entropy weighting is an objective weighting method, which is based on the principle that the smaller the degree of variability of an indicator and the less information it can reflect, the lower its corresponding weight (Jiang et al., 2022a). With *m* samples; *n* specific rating indicators, each indicator *M_ij_* (*i*=1, 2, 3,…, *m*; *j*=1, 2, 3,…, *n*) is normalized to determine the value of information entropy (Jiang et al., 2022a) of each indicator:


(3) $$P_{ij}=N_{ij}/\sum_{i=1}^{m}N_{ij}$$

Where *P_ij_* is the contribution value of sample *M_ij_*; *N_ij_* is the normalized value.


(4) $$S_{j}=-X\sum_{i=1}^{m}\left[P_{ij}\times \ln(P_{ij})\right]$$

Where *S_j_* is the value of information entropy; the constant *X*=1/ln(*m*), making 0≤*S_j_ ≤* 1; that is, the maximum of *S_j_* is 1. The smaller *S_j_* is, the greater the significance of the indicator for the whole.


(5) $$Q_{j}=1-S_{j}$$

(6) $$T_{j}=(1-S_{j})/\sum_{j=1}^{n}\left(1-S_{j}\right)$$

Where *Q_j_* is the value of information utility; *T_j_* is the weight (%) obtained for each indicator.

(2) Multicriteria assessment:

In order to comprehensively evaluate the overall performance of different treatment options, a standardized evaluation system of 0–10 grades was constructed using the minimum mean (*Min*) and maximum mean (*Max*) of the total irrigation volume, water consumption, crop yield and quality of the intercropping system as the evaluation indexes, where 0 is the worst performance and 10 is the best performance (Wang et al., 2023c; Xu et al., 2022). The crop yield and quality of the intercropping system were calculated by the following equations (Wang et al., 2023c):


(7) Scale=10×(Value-Min)/(Max-Min)

Irrigation and consumption in the intercropping system were calculated by the following equations:


(8) Scale=10-[10×(Value-Min)/(Max-Min)]

### 2.4 Data analysis

Microsoft Excel 2010 (Microsoft Corp., Raymond, Washington, USA) was used for data organization and multicriteria assessment. Analysis of variance (ANOVA) was performed by utilizing IBM SPSS Statistics 25.0 software (IBM, Inc., New York, USA). Analysis of variance (ANOVA) and multiple comparisons were performed using one-way (One-way ANOVA) and Duncan’s method (*P*< 0.05), and analysis of water regulation and cropping pattern and their interaction effects were performed using two-way ANOVA (*P*< 0.05). Plotting was performed using Origin 2021 (Origin Lab, Corp., Hampton, Massachusetts, USA) software.

## 3 Results

### 3.1 Effect of water regulation on crop growth of wolfberry-alfalfa intercropping system

#### 3.1.1 Wolfberry growth

**Figure 3:**
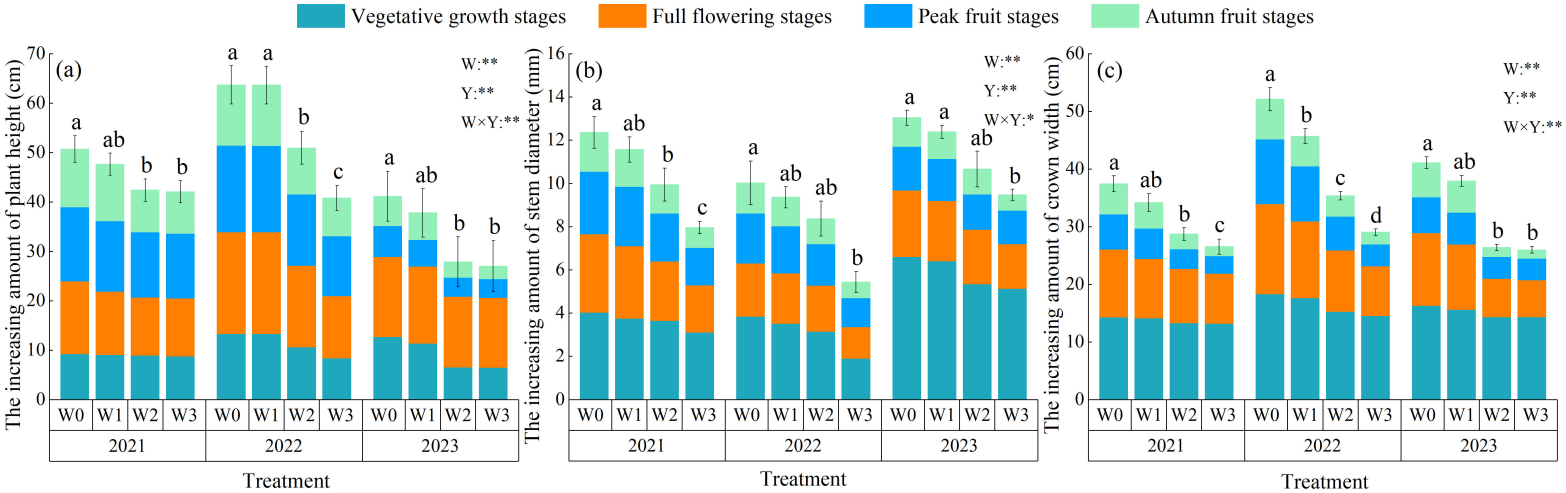
Effects of water regulation on the growth of wolfberry in wolfberry-alfalfa intercropping system. **(a, c)** represents the increasing amount of wolfberry plant height, stem diameter and crown width, respectively. The W0, W1, W2 and W3 refer to full irrigation (75%–85%*θ_f_*), mild water deficit (65%–75%*θ_f_*), moderate water deficit (55%–65%*θ_f_*) and severe water deficit (45%–55%*θ_f_*), respectively. Different lowercase letters indicate the differences among different treatments in the same year. The Y indicates planting year, W indicates water regulation, and Y × W is the interaction of the two; ** indicates highly significant difference (*P*< 0.01), * indicates significant difference (*P*< 0.05), and ns indicates not significant (*P* > 0.05).

Water regulation and planting years highly significantly affected the plant height, stem diameter and crown width of wolfberry (*P*< 0.01), and the interaction of the two highly significantly affected the plant height and crown width of wolfberry (*P*< 0.01), and had a significant effect on the stem diameter growth of wolfberry (*P*< 0.05, **Figure 3**). The increasing amount of wolfberry plant height, stem diameter and crown width throughout the reproductive period decreased with the intensification of water deficit. With the advancement of the fertility period of wolfberry, the increasing amount of wolfberry plant height under the control of each water increased first and then decreased, and reached the maximum at the full flowering stage. The increasing amount of wolfberry stem diameter and crown width decreased and reached the maximum at the vegetative growth stage. The increasing amount of wolfberry plant height in the 3-year growing seasons was 42.06–50.68 cm (2021), 40.79–63.69 cm (2022), and 27.02–41.15 cm (2023), all of which showed an increase of 20.49%–56.14%, 13.27%–56.04%, and 0.80%–24.87% in W0, W1, and W2 treatments compared with the W3 treatment, respectively; and the increasing amount of wolfberry stem diameter was 7.96–12.36 mm (2021), 5.43–10.02 mm (2022), and 9.47–13.04 mm (2023), compared with the W3 treatment, W0, W1 and W2 treatments increased 37.71%–84.59%, 30.81%–72.38% and 12.61%–54.21%, respectively; the increasing amount of wolfberry crown width increased 26.54–37.42 cm (2021), 29.05–52.12 cm (2022) and 25.99–41.07 cm (2023). W0, W1 and W2 treatments increased by 41.03%–79.45%, 28.88%–57.25% and 1.62%–21.75%, respectively, compared with W3 the treatment.

#### 3.1.2 Alfalfa growth

**Figure 4:**
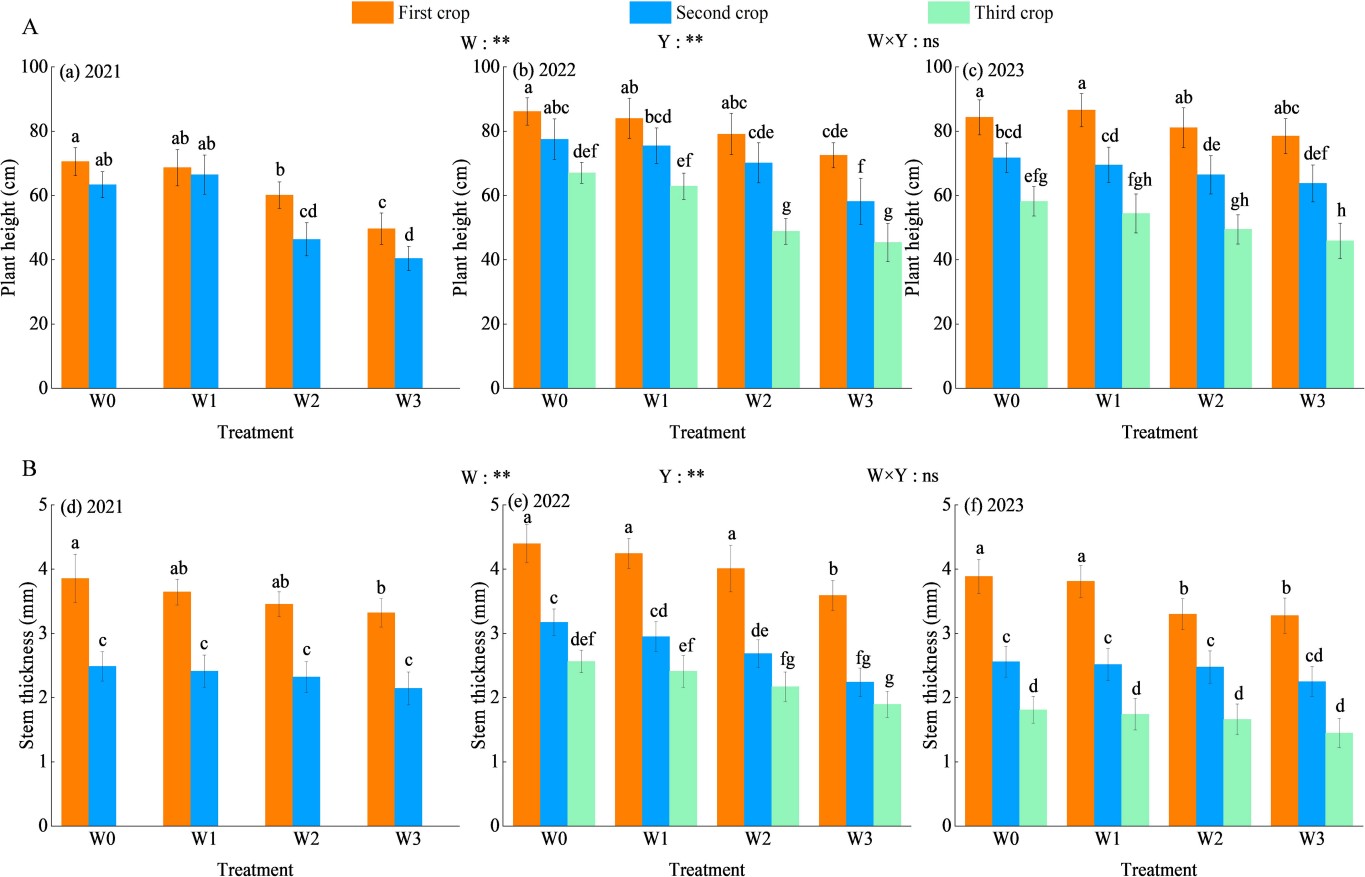
Effect of water regulation on the growth of alfalfa in wolfberry-alfalfa intercropping system. **(A, B)** represent alfalfa plant heights and stem thickness. (a–f) represent alfalfa growth indexes in 2021, 2022 and 2023, respectively. The W0, W1, W2 and W3 refer to full irrigation (75%–85%*θ_f_*), mild water deficit (65%–75%*θ_f_*), moderate water deficit (55%–65%*θ_f_*) and severe water deficit (45%–55%*θ_f_*), respectively. Different lowercase letters indicate the differences among different treatments in the same year. Y indicates planting year, W indicates water regulation, and Y × W is the interaction of the two; ** indicates highly significant difference (*P*< 0.01), and ns indicates not significant (*P* > 0.05).

Water regulation and planting years highly significantly affected alfalfa plant height (*P*< 0.01), but the interaction between the two had no significant effect (*P* > 0.05, **Figure 4A**). The plant height of alfalfa increased with the advance of planting years and decreased with the intensification of water deficit and the advance of stubble times. Compared with 2021, the plant height under the four kinds of water regulation in 2022 and 2023 increased by 9.70%–30.31% and 3.83%–39.21% on average. The overall performance of alfalfa plant height in the 3-year growing season was W0 > W1 > W2 > W3, with an average increase of W0, W1 and W2 treatments was 13.91%–48.65%, 11.90%–50.04% and 4.74%–18.21%, respectively, compared with the W3 treatment.

Water regulation and planting years highly significantly affected alfalfa stem thickness (*P*< 0.01), but the interaction between the two had no significant effect (*P* > 0.05, **Figure 4B**). The stem thickness of alfalfa increased first and then decreased with the advance of planting years, and decreased with the intensification of water deficit and the advance of stubble times. Compared with 2021 and 2023, the stem thickness of alfalfa under four kinds of water regulation increased by 0.07%–6.55% and 10.75%–22.76% on average in 2022. In the 3-year growing season, the overall performance of alfalfa stem thickness was W0 > W1 > W2 > W3, and the average increase of W0, W1 and W2 treatments was 16.05%–31.15%, 10.86%–24.29% and 5.67%–14.75% compared with the W3 treatment, respectively.

### 3.2 Effects of water regulation on crop physiology of wolfberry-alfalfa intercropping system

#### 3.2.1 Wolfberry physiology

**Figure 5:**
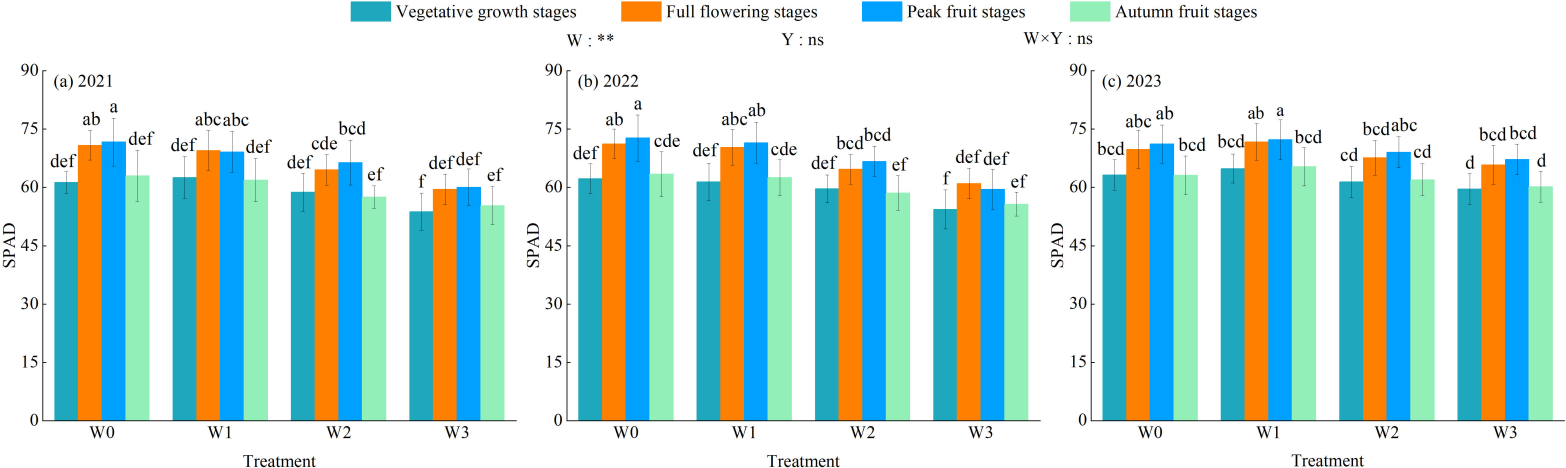
Effect of water regulation on SPAD of wolfberry in wolfberry-alfalfa intercropping system. **(a – c)** represents SPAD of wolfberry in 2021, 2022 and 2023, respectively. The W0, W1, W2 and W3 refer to full irrigation (75%–85%*θ_f_*), mild water deficit (65%–75%*θ_f_*), moderate water deficit (55%–65%*θ_f_*) and severe water deficit (45%–55%*θ_f_*), respectively. Different lowercase letters indicate the differences among different treatments in the same year. Y indicates planting year, W indicates water regulation, and Y × W is the interaction of the two; ** indicates highly significant difference (*P*< 0.01), and ns indicates not significant (*P* > 0.05).

Water regulation extremely significantly affected the chlorophyll content of wolfberry leaves (SPAD, *P<* 0.01), but planting years and their interaction had no significant effects (*P* > 0.05, **Figure 5**). The SPAD of wolfberry leaves decreased with the intensification of water deficit. With the advancement of wolfberry fertility period, the SPAD of wolfberry leaves under each water regulation showed a trend of increasing and then decreasing, and reached the maximum at the peak fruit stage. The SPAD of wolfberry in the 3-year growing season was 57.16–66.69 (2021), 57.63–67.39 (2022), and 63.18–68.54 (2023), under different water regulation, the maximum SPAD values of leaves at vegetative growth stage, full flowering stage, peak fruit stage and autumn fruit stage all appeared in W0 treatment. The W0, W1 and W2 treatments increased by 5.76%–16.93%, 8.48%–15.29% and 2.90%–8.22%, respectively, compared with the W3 treatment.

**Figure 6:**
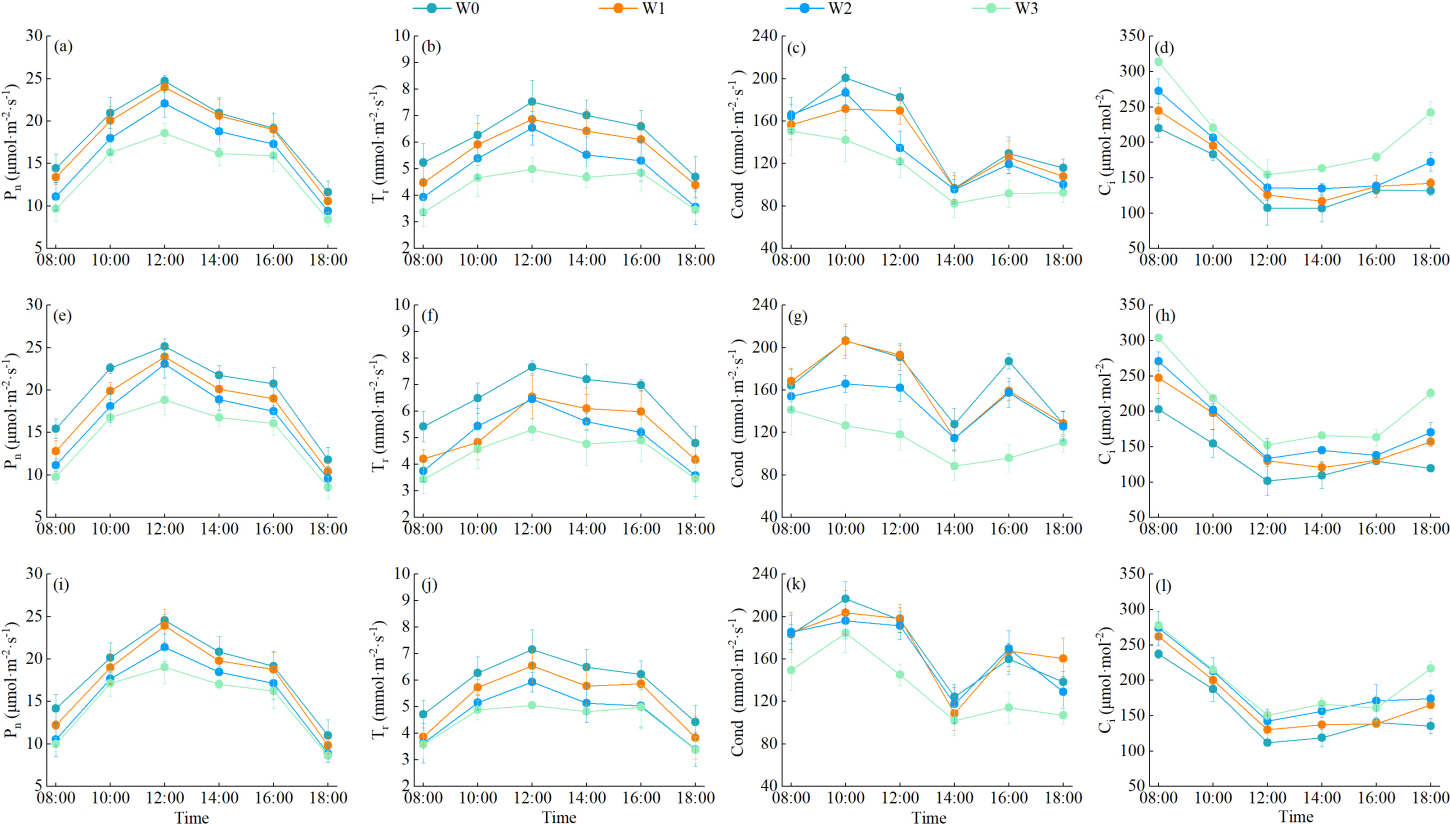
Effect of water regulation on photosynthetic characteristics of wolfberry in wolfberry-alfalfa intercropping system. **(a–l)** in the figure represent the photosynthetic indexes of wolfberry in 2021, 2022 and 2023, respectively. P_n_ represents net photosynthetic rate, T_r_ represents transpiration rate, Cond represents stomatal conductance, and C_i_ represents intercellular carbon dioxide concentration.

The characteristics of daily changes of photosynthetic indexes of wolfberry under different water regulations were shown in **Figure 6**. In the 3-year growing season, the daily changes of net photosynthetic rate (P_n_) and transpiration rate (T_r_) of wolfberry leaves basically showed a single-peak curve, the peak value appeared at 12:00. The first peak of stomatal conductance (Cond) within a day appeared at 10:00, then decreased, then increased slightly after 14:00, and reached the second peak at 16:00, and thereafter showed a downward trend. Intercellular carbon dioxide concentration (C_i_) showed a “V” shape during the day, and the intraday lowest value occurred between 12:00 and 14:00. With the aggravation of the degree of water deficit, the average daily P_n_, T_r_ and Cond of wolfberry leaves showed a trend of decreasing, while C_i_ showed a trend of increasing. The daily average P_n_, T_r_ and Cond of wolfberry leaves were greatest under W0 treatment, which increased by 3.98%–35.29%, 9.27%–46.51% and 0.34%–47.54%, respectively, compared with other treatments; while C_i_ was greatest under W3 treatment, which increased by 4.95%–50.38% compared with other treatments.

#### 3.2.2 Alfalfa physiology

**Figure 7:**
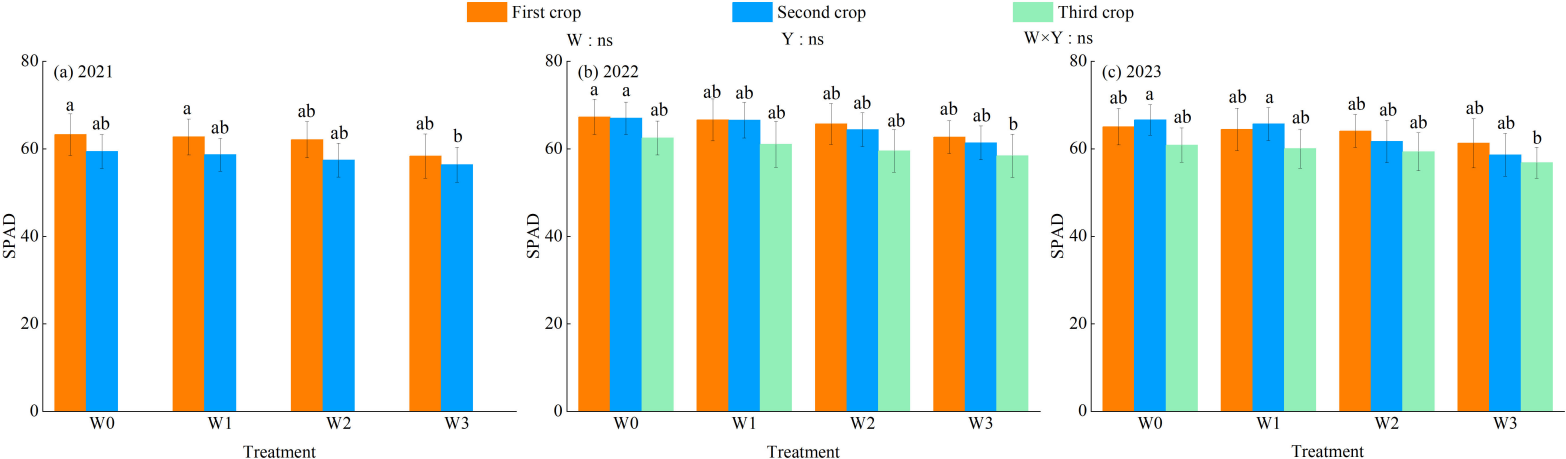
Effect of water regulation on SPAD of alfalfa in wolfberry-alfalfa intercropping system. **(a – c)** represents SPAD of alfalfa in 2021, 2022 and 2023, respectively. The W0, W1, W2 and W3 refer to full irrigation (75%–85%*θ_f_*), mild water deficit (65%–75%*θ_f_*), moderate water deficit (55%–65%*θ_f_*) and severe water deficit (45%–55%*θ_f_*), respectively. Different lowercase letters indicate the differences among different treatments in the same year. Y indicates planting year, W indicates water regulation, and Y × W is the interaction of the two; ns indicates not significant (*P* > 0.05).

The effects of water regulation, planting years and the interaction between the two on chlorophyll content (SPAD) of alfalfa leaves were not significant (*P* > 0.05, **Figure 7**). The SPAD of alfalfa leaves decreased with the increase of water deficit. As alfalfa stubble advanced, the SPAD of alfalfa leaves showed a decreasing trend under each water regulation, and the SPAD of alfalfa leaves in the first stubble was the largest (except for W0 and W1 treatments in 2023). In the 3-year growing season, the SPAD values of alfalfa were 57.37–61.34 (2021), 60.82–65.59 (2022) and 58.92–64.19 (2023). The maximum SPAD values of alfalfa leaves under different water regulations all appeared in the W0 treatment. The W0, W1 and W2 treatments increased by 6.92%–8.94%, 5.80%–7.59% and 3.90%–4.72%, respectively, compared with the W3 treatments.

**Figure 8:**
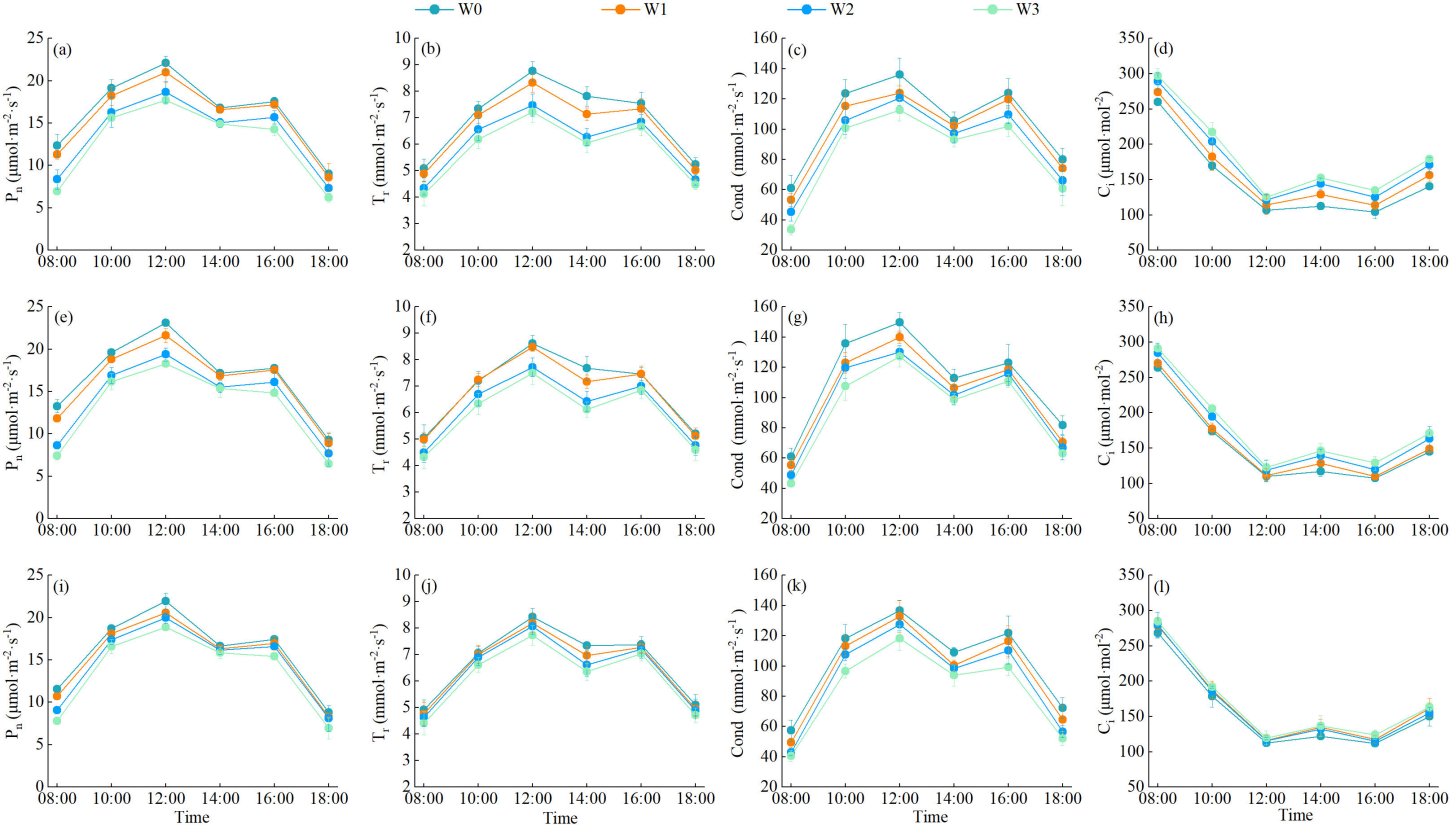
Effect of water regulation on photosynthetic characteristics of alfalfa in wolfberry-alfalfa intercropping system. **(a–l)** in the figure represent the photosynthetic indexes of alfalfa in 2021, 2022 and 2023, respectively. P_n_ represents net photosynthetic rate, T_r_ represents transpiration rate, Cond represents stomatal conductance, and C_i_ represents intercellular carbon dioxide concentration.

The characteristics of daily changes in photosynthetic indexes of alfalfa under different water regulations are shown in **Figure 8**. In the 3-year growing season, the daily changes in net photosynthetic rate (P_n_), transpiration rate (T_r_) and stomatal conductance (Cond) of alfalfa leaves basically showed a double-peak curve, the first peak appeared at 12:00, showed a downward trend, and then showed a small upward trend after 14:00, and the second peak reached at 16:00, and then showed a downward trend. The intercellular carbon dioxide concentration (C_i_) showed a “V” shape during the day, and the intra-day lowest value occurred between 12:00 and 16:00. With the aggravation of the degree of water deficit, the average daily P_n_, T_r_ and Cond of wolfberry leaves showed a tendency to decrease, while C_i_ showed a trend of increasing. The daily average P_n_, T_r_ and Cond of alfalfa leaves were greatest under W0 treatment, increasing by 4.32%–28.07%, 1.88%–20.32% and 6.64%–25.42%, respectively, compared with other treatments, while C_i_ was greatest under W3 treatment, increasing by 2.46%–23.83% compared with other treatments.

### 3.3 Effects of water regulation on crop yield of wolfberry-alfalfa intercropping system

Water regulation and planting years highly significantly affected the irrigation amount, water consumption (ET) (Equation 1), dried wolfberry fruit yield, fresh wolfberry fruit yield and total alfalfa yield in the wolfberry-alfalfa system (*P*< 0.01), and the interaction of the two highly significantly affected the irrigation amount, dried wolfberry fruit yield, fresh wolfberry fruit yield and total alfalfa yield (*P*< 0.01, **Table 1**). With the increase of planting years, the irrigation amount, ET, dried wolfberry fruit yield (except W3 treatment) and the total alfalfa yield (except W0 treatment) under each water regulation showed an increasing trend, and the alfalfa yield showed a decreasing trend with the advancement of the crop. As the degree of water deficit increased, the yield of dried wolfberry showed a decreasing trend, reaching the maximum in W0 treatment, which was 2648.79 kg·ha^−1^ and 2711.82 kg·ha^−1^; the total alfalfa yield in 2021 and 2022 reached the maximum in W0 treatment, which was increased by 4.37%–22.50% and 5.92%–26.69% compared with other treatments, whereas in 2023, total alfalfa yield was maximized in W1 treatment, which was increased by 5.66%–12.96% compared with other treatments, respectively.

**Table 1:** Effect of water regulation on crop yield in wolfberry-alfalfa intercropping system.

Year	Treatment	Irrigation amount (m^3^·ha^-1^)	ET (m^3^·ha^-1^)	Yield (kg·ha^-1^)					
				Wolfberry		Alfalfa			
				Dry weight	Fresh weight	First crop	Second crop	Third crop	Total yield
2021	W0	4125.60a	5123.00a	—	—	1820.30a	1589.77a	—	3410.08a
	W1	3755.61b	4520.31b	—	—	1738.12a	1529.25ab	—	3267.38a
	W2	3152.41c	3845.60c	—	—	1621.73b	1380.55b	—	3002.28b
	W3	2210.10d	3256.90d	—	—	1598.04b	1185.75c	—	2783.79c
2022	W0	4594.00a	5363.91a	2648.79a	9238.62a	7453.81a	3791.17a	2382.71a	13627.68a
	W1	3904.90b	4748.42b	2535.68a	9163.95a	7004.02b	3598.39ab	2263.55a	12865.96b
	W2	3215.81c	4130.90c	2123.21b	7703.91b	6682.73b	3341.37bc	1928.34a	11952.44c
	W3	2426.71d	3616.11d	1682.04c	5908.07c	5847.39c	3212.85c	1696.36a	10756.60d
2023	W0	5192.00a	5678.41a	2711.82a	9427.04a	5234.00a	4687.38a	3395.63a	13317.02a
	W1	4113.21b	4819.80b	2671.00a	9458.69a	5573.01ab	4740.03a	3757.69a	14070.72ab
	W2	3634.40c	4343.30c	2288.78b	8375.17b	4940.00b	4326.93b	3243.16a	12510.09b
	W3	2855.60d	3767.00d	1588.20c	5481.71c	4931.04b	4281.64b	3243.70a	12456.34b
Analysis of variance									
Y		**	**	**	**	**	**	**	**
W		**	**	**	**	**	**	ns	**
Y×W		**	ns	**	**	**	ns	ns	**

The W0, W1, W2 and W3 refer to full irrigation (75%–85%*θ_f_*), mild water deficit (65%–75%*θ_f_*), moderate water deficit (55%–65%*θ_f_*) and severe water deficit (45%–55%*θ_f_*), respectively. ET indicates water consumption. Different lowercase letters indicate the differences among different treatments in the same year. Y indicates planting year, W indicates water regulation, and Y × W is the interaction of the two; ** indicates highly significant difference (*P*< 0.01), and ns indicates not significant (*P* > 0.05).

### 3.4 Effects of water regulation on crop quality of wolfberry-alfalfa intercropping system

#### 3.4.1 Wolfberry quality

**Figure 9:**
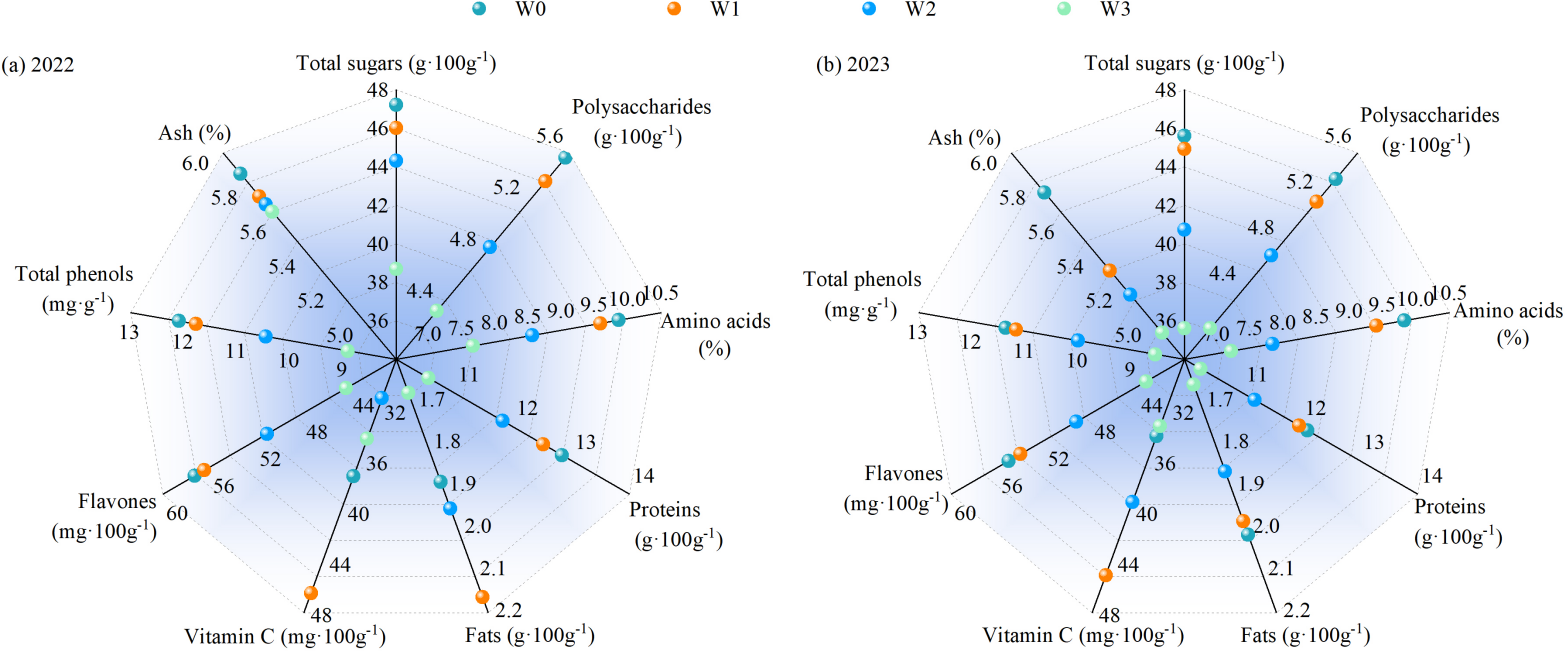
Effect of water regulation on quality of wolfberry in wolfberry-alfalfa intercropping system. **(a, b)** represents the quality of wolfberry in 2022 and 2023, respectively. The W0, W1, W2 and W3 refer to full irrigation (75%–85%*θ_f_*), mild water deficit (65%–75%*θ_f_*), moderate water deficit (55%–65%*θ_f_*) and severe water deficit (45%–55%*θ_f_*), respectively.

Water regulation significantly affected the quality of wolfberry (*P*< 0.05, except crude ash content), and planting years significantly affected the contents of total sugar, polysaccharide and flavonoid (*P*< 0.05), but had no significant effect on other quality indexes of wolfberry (*P* > 0.05), and the interaction of the two had no significant effect on the quality of wolfberry (*P* > 0.05, **Table 2**). In the growing season of 2022, the total sugar content, polysaccharides content, amino acids content, proteins content, flavones content, total phenols content and ash content all decreased with the increase of water deficit, while the fats content and vitamin C content reached the maximum in the W1 treatment, which were expressed as W1 > W2 > W0 > W3 and W1 > W0 > W3 > W2, respectively (**Figure 9**). Compared with the growing season of 2022, the quality of wolfberry under four kinds of water regulation decreased in the growing season of 2023, and the total sugar content, polysaccharide content, amino acid content, protein content, fat content, flavone content, total phenol content and ash content all decreased with the intensification of the degree of water deficit, and the basic performance was W0 > W1 > W2 > W3. The content of vitamin C was W1 > W2 > W0 > W3.

**Table 2:** Difference significance analysis of different wolfberry quality in the wolfberry-alfalfa intercropping system.

Index	Y	W	Y×W
Total sugars content	**	**	ns
Polysaccharides content	*	**	ns
Amino acids content	ns	**	ns
Proteins content	ns	**	ns
Fats content	ns	**	ns
Vitamin C content	ns	**	ns
Flavones content	*	**	ns
Total phenols content	ns	*	ns
Ash content	ns	ns	ns

Y indicates planting year, W indicates water regulation, and Y × W is the interaction of the two; ** indicates highly significant difference (*P*< 0.01), * indicates significant difference (*P*< 0.05), and ns indicates not significant (*P* > 0.05).

#### 3.4.2 Alfalfa quality

**Figure 10:**
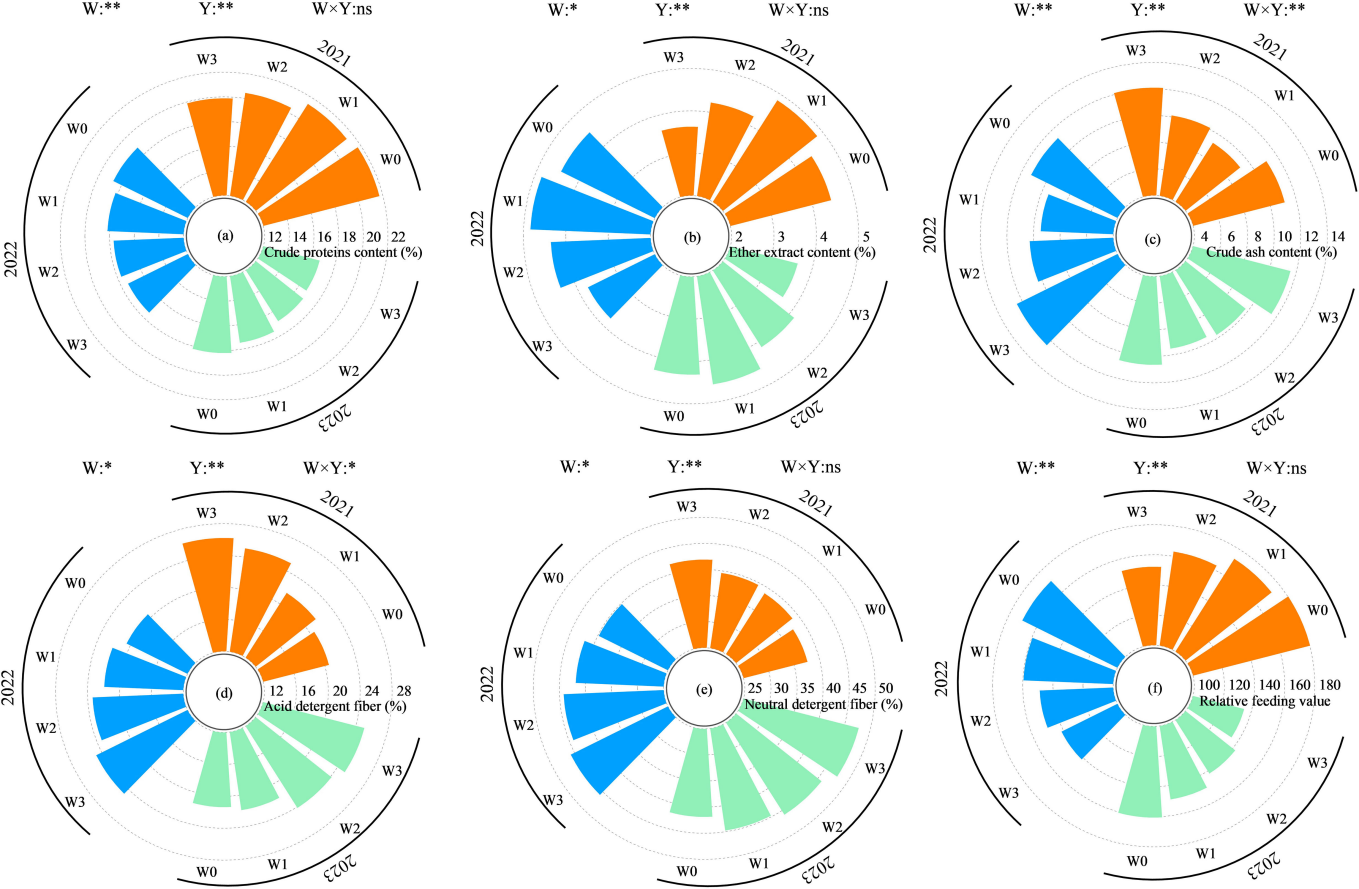
Effect of water regulation on quality of alfalfa in wolfberry-alfalfa intercropping system. **(a–f)** respectively represent the annual crude protein content, ether extract content, crude ash content, acid detergent fiber content, neutral detergent fiber content and relative feeding value of alfalfa. The W0, W1, W2 and W3 refer to full irrigation (75%–85%θ_*f*_), mild water deficit (65%–75%θ_*f*_), moderate water deficit (55%–65%θ_*f*_) and severe water deficit (45%–55%θ_*f*_), respectively. Y indicates planting year, W indicates water regulation, and Y × W is the interaction of the two; ** indicates highly significant difference (*P*< 0.01), * indicates significant difference (*P*< 0.05), and ns indicates not significant (*P* > 0.05).

Water regulation and planting years significantly affected alfalfa quality (*P*< 0.05), and the interaction between the two had a significant effect on alfalfa crude ash and acid detergent fiber content (*P*< 0.05), while the effect on other alfalfa quality indexes were not significant (*P* > 0.05, **Figure 10**). With the intensification of water deficit, the alfalfa crude protein content and relative feeding value (Equation 2) showed a decreasing trend during the 3 years; crude fat content showed an increasing and then decreasing trend; and acid detergent fiber and neutral detergent fiber content showed a decreasing trend. In the growing season of 2021, the crude protein content, crude fat content, crude ash content, acid detergent fiber content, neutral detergent fiber content and relative feeding value of alfalfa under the four kinds of water regulation were 19.89%–21.72%, 3.63%–4.84%, 9.24%–12.10%, 20.51%–26.21%, 37.66%–41.90%, and 152.05%–172.87%, respectively. The crude protein content of the W3 treatment was reduced by 8.44%, 7.09%, and 2.74% compared with the W0, W1, and W2 treatments, and the crude fat content was reduced by 18.56%, 25.08% and 14.25%, and the crude ash content was increased by 8.90%, 30.94% and 19.65%, and the acid detergent fiber content was increased by 27.76%, 21.29% and 4.43%. The neutral detergent fiber content was increased by 11.27%, 8.02% and 5.89%, and the relative feeding value was reduced by 15.63%, 12.04% and 6.77%. After 3 years of intercropping, the quality of alfalfa decreased, and the crude protein content and relative feeding value decreased by 16.98% and 12.17% on average, while the acid detergent fiber and neutral detergent fiber increased by 1.01% and 14.22%, respectively.

### 3.5 Entropy weight–multicriteria assessment

The entropy weight method can eliminate the interference of human factors and has the advantage of objective weighting. The entropy weight method was used to select the most representative quality indicators of wolfberry and alfalfa for comprehensive evaluation (Equations 3–6). As can be seen from **Table 3** and **Table 4**, the sensitivity of quality indicators of wolfberry to water regulation was ranked as follows: Vitamin C content > ash content > amino acid content > polysaccharide content > total phenol content > flavone content > protein content > total sugar content > crude fat content. The sensitivity of alfalfa quality indicators to water regulation was ranked as: crude protein content > crude ash content > acid detergent fiber content > relative feeding value > neutral detergent fiber content > crude fat content. The weight coefficients of the vitamin C content of wolfberry and crude protein content of alfalfa were 14.23% and 11.56%, respectively. Therefore, they were used as important indicators for evaluating the crop quality of the wolfberry-alfalfa intercropping system for the optimal water regulation program preference.

**Table 3:** Optimization of quality index of wolfberry based on entropy weight method.

Parameter	Total sugars	Polysaccharides	Amino acids	Proteins	Fats	Vitamin C	Flavones	Total phenols	Ash
Information entropy value (S_j_)	0.4888	0.4778	0.4572	0.4856	0.4888	0.3023	0.4832	0.4819	0.4300
Information utility value (Q_j_)	0.5112	0.5222	0.5428	0.5144	0.5112	0.6977	0.5168	0.5181	0.5700
Weight coefficient (T_j_,%)	10.4232	10.6475	11.0680	10.4898	10.4227	14.2262	10.5369	10.5633	11.6225

**Table 4:** Optimization of quality index of alfalfa based on entropy weight method.

Parameter	Crude proteins content	Ether extract content	Crude ash content	Acid detergent fiber	Neutral detergent fiber	Relative feeding value
Information entropy value (S_j_)	0.4328	0.4841	0.4402	0.4444	0.4637	0.4551
Information utility value (Q_j_)	0.5672	0.5159	0.5598	0.5556	0.5363	0.5449
Weight coefficient (T_j_,%)	11.5646	10.5202	11.4154	11.3286	10.9361	11.1102

**Figure 11:**
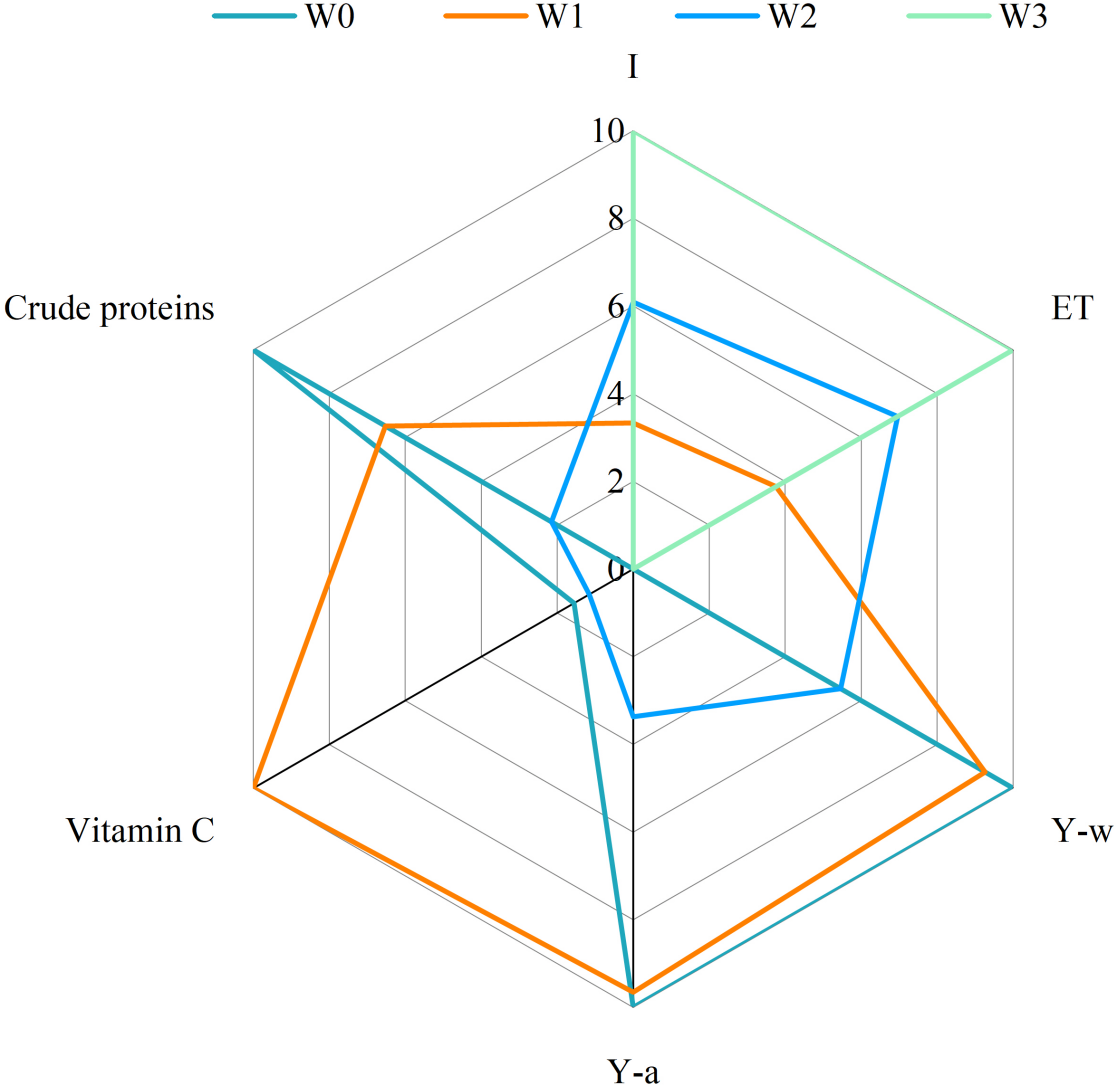
Multicriteria assessment of treatments. Y-w indicates wolfberry yield, Y-a indicates alfalfa yield, I indicates irrigation, and ET indicates water consumption.

Irrigation (I), water consumption (ET), wolfberry yield (Y-w), alfalfa yield (Y-a), wolfberry quality (vitamin C content) and alfalfa quality (crude protein content) under different water regulation treatments were selected for multicriteria assessment (Equations 7–8). As can be seen from **Figure 11**, the total crop yield and wolfberry quality scores of the intercropping system under W0 treatment were higher, while the scores of I and ET were lower, with an average score of 5.26; in contrast, the W2 and W3 treatments scored higher for I and ET, while the total system crop yield and quality scored lower, with average scores of 4.20 and 3.33, respectively. The W1 treatment showed the best balance on all indicators and had the highest average score (7.09). The comprehensive scoring results indicated that mild water deficit treatment (W1) could effectively realize the synergistic efficiency of the wolfberry-alfalfa intercropping system, and significantly enhance the yield and quality of the system while ensuring the efficient utilization of water.

## 4 Discussion

### 4.1 Effect of water regulation on crop growth in wolfberry-alfalfa intercropping system

The good growth and development of crops are the key to the synergistic improvement of yield and quality, and water is an important environmental factor to ensure crop growth and development, which directly affects crop photosynthesis, nutrient absorption and metabolic activities (Seidel et al., 2017). Appropriate water regulation can promote healthy crop growth, improve yield and quality. Amanullah et al. (2021) found in the cereal-legume intercropping system that the growth rate of each crop in the intercropping system was higher under sufficient irrigated condition than deficit irrigation treatment. In this study, we also found that in the wolfberry-alfalfa intercropping system, the increasing amount of wolfberry plant height, stem diameter and crown width decreased with the increasing degree of water deficit, and the plant height and stem thickness of alfalfa showed the same changing trend. This may be because sufficient irrigation provided adequate water for both wolfberry and alfalfa, which enabled better root growth and enhanced root respiration and nutrient absorption, thus promoting crop growth and development (Khan et al., 2024). However, Zheng et al. (2021) found that the length of apple new shoots gradually increased with the increase of irrigation water, while the height of soybean plants showed a trend of increasing and then decreasing in the Loess Plateau region in a study of a 5-year-old apple-soybean intercropping system under straw mulching. This is different from the result of optimal crop growth of wolfberry-alfalfa system under sufficient irrigated conditions in this study, which may be due to the fact that both wolfberry and alfalfa are perennial plants, and their deep root systems can reach deep soil moisture, but the conservative resource allocation strategy formed by long-term evolution makes them more sensitive to short-term water deficit. In contrast, soybean, as an annual crop, has a rapid response mechanism in its root system, which can improve water uptake and utilization efficiency under appropriate water stress by increasing root-crown ratio and promoting lateral root differentiation. Through the adjustment of root system structure, soybean can supply limited water to the critical reproductive period, thus realizing the compensatory growth effect under suitable water deficit conditions (Battisti et al., 2017). In addition, this study found that the increasing amount of wolfberry stem diameter and crown width reached the maximum at the full flowering stage, and the alfalfa plant height and stem thickness obtained the maximum value at the first crop. This may be due to the fact that the full flowering stage is the peak period of nutrient growth of wolfberry, and nutrients are mainly used for branch and leaf growth and crown expansion, and the growth of stem diameter and crown width increased significantly; alfalfa in the growth stage of the first crop, the soil nutrients and water were sufficient, which was more conducive to its own development, and the growth of the subsequent crops was limited by the reduction of soil nutrients and the consumption of its own growth.

### 4.2 Effect of water regulation on crop physiology in wolfberry-alfalfa intercropping system

Photosynthesis is an important physiological process in the plant life cycle, which converts CO_2_ and H_2_O into organic matter by absorbing light energy, directly affecting crop yield and quality (Dong et al., 2024; Flexas and Carriquí, 2020). Water is the basis of crop photosynthesis, and sufficient water can keep leaf stomata open and promote CO_2_ absorption and light energy conversion. Appropriate water conditions not only improve photosynthetic efficiency but also enhance crop growth potential and stress resistance, which is crucial for the sustainability of agricultural production. This study found that in the wolfberry-alfalfa intercropping system, the SPAD values of both wolfberry and alfalfa leaves decreased with increasing water deficit, which may be due to the fact that water deficit reduced soil nutrient availability, especially nitrogen absorption and utilization, and chlorophyll synthesis depends on nitrogen, and insufficient supply of nutrients directly affected chlorophyll content, which in turn reduced the SPAD values (Liang et al., 2023). The present study showed that daily average P_n_, T_r_ and Cond of wolfberry and alfalfa leaves tended to decrease with increasing water deficit. However, Wang et al. (2023a) found in an apple-soybean intercropping system in the Loess Plateau region that apple P_n_ was highest under sufficient irrigated (80% FC) conditions, whereas apple T_r_ and soybean P_n_ and T_r_ were both highest under mild water deficit (65% FC) conditions. This difference may be due to the damage of the chlorophyll structure of crop leaves by water deficit, resulting in pigment decomposition, lower SPAD, and reduced CO_2_ solubility in leaf mesophyll cells, which in turn reduced the photosynthetic rate (Xin et al., 2023). At this point, wolfberry tended to close stomata to minimize leaf water loss, thus inhibiting photosynthesis. Meanwhile, in this study, the drip irrigation belt was close to wolfberry, and alfalfa would preferentially maintain its survival rather than growth by absorbing deep water, so the accumulation of photosynthetic products was suppressed. In addition, it was found that the daily changes of P_n_ and T_r_ in wolfberry leaves showed a single-peak curve, which peaked at 12:00; while the daily changes of P_n_ and T_r_ in alfalfa leaves showed a double-peak curve, which peaked at 12:00 and 16:00, respectively; and the intra-day changes of C_i_ in wolfberry and alfalfa leaves showed a “V”-shape, with the lowest values occurring from 12:00 to 16:00. This result was inconsistent with the study of Zheng et al. (2021) in an apple-soybean intercropping system, who found that the phenomenon of “midday depression of photosynthesis” did not exist in the intercropping system, and that the daily variations of P_n_ and T_r_ showed a single-peak curve. The reason for this discrepancy may be that wolfberry experienced a short “midday depression of photosynthesis”, when the solar zenith angle reached its maximum value at noon, the photosynthetic rate of wolfberry leaves gradually decreased. Although the solar zenith angle gradually decreased afterwards, there was still accumulated temperature on the ground, which was relatively high. In addition, the growth period of wolfberry was long, and the root system did not provide timely water supply, resulting in the loss of leaf water that could not be replenished in a timely manner, and the photosynthetic rate was difficult to recover. Therefore, the second peak did not appear (Li et al., 2021). However, alfalfa exhibited a “midday depression of photosynthesis” phenomenon from 12:00-15:00. The high temperature at noon caused the stomata to close and the photosynthetic rate to decrease. After 15:00, as the temperature decreased, stomata may reopen, and the shading effect of wolfberry promoted the appearance of the second peak of alfalfa photosynthetic rate.

### 4.3 Effect of water regulation on crop yield and quality in wolfberry-alfalfa intercropping system

Appropriate water regulation is key to high crop yield. Optimal spatial and temporal allocation of water supply during the crop growth period not only enhances water-nutrient coupling absorption at the root-soil interface but also significantly improves the efficiency of photosynthetic product accumulation by regulating the source-sink relationship (Jia et al., 2017; Tang et al., 2010). This study found through a 3-year term experiment, that the yield of wolfberry dried fruit decreased with the intensification of water deficit, while the yield of alfalfa showed a parabolic response curve, first increasing and then decreasing. The possible reasons are that, on the one hand, due to the high dependence of reproductive growth of wolfberry on water, and the positive response to water regulation, water stress will significantly inhibit the stomatal conductance of wolfberry leaves, resulting in a decrease in the rate of CO_2_ assimilation, which directly affects the accumulation of photosynthetically produced products, so wolfberry has the highest yield under the condition of full irrigation; on the other hand, after 3 years of intercropping, soil water and nutrients gradually accumulated in deeper layers, and alfalfa, with its deep root system, could more effectively utilize deep soil resources (Song et al., 2022). Mild water deficit stimulated alfalfa’s physiological resilience, optimized inter-species resource allocation, and then increased yield. Additionally, this study found that wolfberry dried fruit yield and alfalfa yield under all water regulation treatments increased with planting duration, which is similar to Dou et al. (2022) on the apple-soybean intercropping system. This is mainly attributed to two aspects: first, after 3 years of intercropping, the system stability and diversity were enhanced, which helped improve the growth potential and yield of crops in the system; second, in the intercropping system, wolfberry and alfalfa formed a virtuous cycle in water and nutrient utilization, ultimately achieving synchronized yield improvement.

Crop quality is an important indicator for measuring production efficiency. Water regulation directly determines crop quality by affecting physiological metabolism and material accumulation. Appropriate water supply can improve crops’ nutritional, flavor, and appearance quality, and enhance market competitiveness (Lu et al., 2019). Ma et al. (2023) concluded from research in arid and semi-arid regions that when irrigation volume was higher (2565–2970 m^3^·ha^−1^), wolfberry’s total sugar content, betaine content, crude fat content, and protein content all reached their highest levels, resulting in optimal wolfberry quality. This study also found that wolfberry’s total sugar content, polysaccharide content, amino acid content, protein content, flavone content, total phenol content, and ash content were all maximized under full irrigation conditions. However, Gao et al. (2024) found that with increasing irrigation, the total sugar and polysaccharide content of wolfberry showed a trend of first increasing and then decreasing, differing from the results of this study. This may be because in the intercropping system, adequate water supply promoted wolfberry’s photosynthesis and metabolic activities, moreover, alfalfa’s root system improved soil structure, increased nutrient supply, and provided nitrogen sources for wolfberry through nitrogen fixation, further promoting efficient utilization of water and nutrients, thereby increasing accumulation of nutritional substances. This study showed that under full irrigation conditions, alfalfa’s crude protein content and relative feed value were maximized, while acid detergent fiber content and neutral detergent fiber content were minimized. However, Varol et al. (2024) found in Turkey that with increasing irrigation levels, alfalfa’s acid detergent fiber, neutral detergent fiber, and crude ash proportions increased, while crude protein proportion decreased. This discrepancy may be that in the wolfberry-alfalfa system, drip irrigation tape was closer to wolfberry, causing water to concentrate in the wolfberry root zone, placing alfalfa in a mild water stress state, which promoted the accumulation of nutritional substances in alfalfa while inhibiting fiber synthesis, thereby improving alfalfa’s feed value (Jiang et al., 2022b). Furthermore, this study also found that the quality of both wolfberry and alfalfa in the intercropping system decreased with increasing intercropping duration. This may be because both wolfberry and alfalfa are perennial crops, and long-term intercropping led to soil nutrient imbalance, changes in soil microbial community structure, and allelopathic effects caused by accumulated root exudates inhibiting crop growth and metabolic activities (He et al., 2023). Meanwhile, the risks of pests, diseases and continuous cropping obstacles have increased, ultimately resulting in decreased quality of wolfberry and alfalfa.

This study revealed the short-term effects of water regulation on the productivity of wolfberry-alfalfa intercropping system based on a 3-year field experiment, but the ecological effects that may be triggered by the long-term continuous application of this water management model still need to be further verified. For example, the synergistic effect of irrigation and intercropping may accelerate the mineralization of soil organic matter and lead to nutrient imbalance in the soil profile due to competitive nutrient uptake by the alfalfa deep root system. In addition, long-term water regulation may alter soil pore structure, which in turn affects microbial community functioning, a secondary effect that may offset the short-term yield-enhancing benefits of intercropping systems. In the future, drones and satellite remote sensing technologies can be utilized to monitor crop growth and pests in real time to improve the efficiency and sustainability of intercropping systems.

## 5 Conclusions

Appropriate water supply can significantly promote crop growth and improve yield and quality in wolfberry-alfalfa intercropping system. Among them, the growth, leaf SPAD, and photosynthetic characteristics of wolfberry and alfalfa were all greatest in the W0 treatment. Over the three-year experiment, W0 and W1 treatments respectively achieved the highest yields for wolfberry (1.53%–70.75% higher) and alfalfa (5.66%–12.96% higher) compared to other treatments. With the increase of planting years, the quality indexes such as crude protein and polysaccharide of the two crops showed a decreasing trend, but they still maintained a relatively optimal level under the W0 treatment. Comprehensive production performance indicators, it is suggested that wolfberry-alfalfa intercropping system combined with 65%–75%*θ_f_* water regulation mode, which can realize efficient utilization of agricultural water resources while ensuring high yield and high quality of crops, and provide theoretical basis and technical support for the sustainable development of agriculture in arid zones of Northwest China.

## Data Availability

The original contributions presented in the study are included in the article/supplementary material. Further inquiries can be directed to the corresponding authors.
